# An assessment on DNA microarray and sequence-based methods for the characterization of methicillin-susceptible *Staphylococcus aureus* from Nigeria

**DOI:** 10.3389/fmicb.2015.01160

**Published:** 2015-10-20

**Authors:** Adebayo O. Shittu, Omotayo Oyedara, Kenneth Okon, Adeola Raji, Georg Peters, Lutz von Müller, Frieder Schaumburg, Mathias Herrmann, Ulla Ruffing

**Affiliations:** ^1^Department of Microbiology, Obafemi Awolowo UniversityIle-Ife, Nigeria; ^2^Institute of Medical Microbiology and Hygiene, Saarland UniversityHomburg, Germany; ^3^Department of Biological Sciences, College of Science, Engineering and Technology, Osun State UniversityOsogbo, Nigeria; ^4^Department of Medical Laboratory Services, Federal Medical CentreMakurdi, Nigeria; ^5^Department of Microbiology and Immunology, Alfaisal UniversityRiyadh, Saudi Arabia; ^6^Institute of Medical Microbiology, University Hospital MünsterMünster, Germany

**Keywords:** *Staphylococcus aureus*, microarray, MLST, genotyping, Nigeria

## Abstract

*Staphylococcus aureus* is an important human pathogen causing nosocomial and community-acquired infections worldwide. In the characterization of this opportunistic pathogen, DNA microarray hybridization technique is used as an alternative to sequence based genotyping to obtain a comprehensive assessment on the virulence, resistance determinants, and population structure. The objective of this study was to characterize a defined collection of *S. aureus* isolates from Nigeria using the microarray technique, and to assess the extent that it correlates with sequence-based genotyping methods. The clonal diversity and genomic content of 52 methicillin-susceptible *Staphylococcus aureus* (MSSA) were investigated by *spa* typing, MLST and DNA microarray hybridization. More than half (55.8%) of these isolates were associated with clonal complexes (CCs) typically associated with methicillin-resistant *S. aureus* (MRSA) clones i.e., CC1, CC5, CC8, CC30, and CC45. Certain genes linked with virulence (*hlgA* and *clfA*) and adherence (*ebpS, fnbA, sspA, sspB*, and *sspP*) were detected in all isolates. A number of genes or gene clusters were associated with distinct clonal types. The enterotoxin gene cluster (*egc*) was linked with CC5, CC25, CC30, CC45, and CC121, enterotoxin H gene (*seh*) with CC1, exfoliative toxin D gene (*etd*) with CC25 and CC80, and the epidermal cell differentiation inhibitor B gene (*edinB*) with CC25, CC80, and CC152. The excellent agreement between data from DNA microarray and MLST in the delineation of Nigerian MSSA isolates indicates that the microarray technique is a useful tool to provide information on antibiotic resistance, clonal diversity and virulence factors associated with infection and disease.

## Introduction

*Staphylococcus aureus* is implicated in a variety of human infections with high rates of morbidity and mortality (Lowy, 1998; Corey, 2009). In infection, *S. aureus* exhibits a coordinated and regulated expression for a wide variety of cell and surface-associated virulence factors (Foster and Höök, 1998; Novick, 2006). These factors mediate adherence to host cells and damaged tissue, facilitate tissue destruction and spreading, promote iron uptake and evasion of host immune system, as well as tissue damage (Skaar and Schneewind, 2004; Grumann et al., 2014). Recent studies in Cameroon (Kihla et al., 2014), Egypt (Ahmed et al., 2014), Gabon (Alabi et al., 2013), Nigeria (Jido and Garba, 2012; Oladeinde et al., 2013), South Africa (Groome et al., 2012; Naidoo et al., 2013), and Tanzania (Kayange et al., 2010; Mhada et al., 2012) have identified *S. aureus* as the main etiological agent for various infections in Africa. Moreover, this species has been recognized as one main cause of community-acquired neonatal sepsis in Africa (Waters et al., 2011). These studies clearly establish the important role of this major human pathogen in tropical Africa.

In many health care institutions in sub-Saharan Africa, the lack of skilled laboratory manpower and resources is a major constraint in the identification of bacterial pathogens from clinical samples. If such analysis can be provided at all, identification of *S. aureus* typically relies on phenotypic methods precluding in-depth strain characterization. Molecular analysis of clonal attribution and presence of single genes contained in *S. aureus* isolates have emerged in pilot studies from select African centers, areas and populations (Ateba Ngoa et al., 2012; Shittu et al., 2012; Seni et al., 2013; Aiken et al., 2014; Egyir et al., 2014; Oosthuysen et al., 2014; Conceição et al., 2015; De Boeck et al., 2015; Kraef et al., 2015; Schaumburg et al., 2015). Nevertheless, in view of the impact of *S. aureus* disease in sub-Saharan Africa, the clonal characterization in concert with a comprehensive analysis of the hitherto ill-described virulence factor armamentarium of *S. aureus* isolates from this region is urgently warranted. Such analyses should target a broad spectrum of variable staphylococcal factors such as genes or gene clusters conferring antibiotic resistance, toxins, virulence, adhesion or immune evasion factors. These analyses have not been performed on a collection of *S. aureus* isolates in Nigeria, and reports from African countries are limited and only addressed a limited and select analytical spectrum (Raji et al., 2013; Aiken et al., 2014; Rovira et al., 2015).

The DNA microarray used for this analysis is a unique and comprehensive genotyping technique based on the analysis of 334 target sequences corresponding to approximately 170 distinct genes and their allelic variants. It enables the simultaneous identification of various gene classes including species markers, genes encoding resistance and virulence properties, exotoxin and adhesion factors, accessory gene regulator (*agr*), capsule, and SCC*mec* types (Monecke et al., 2011). Based on the observation of a high level of genetic diversity from previous investigations on methicillin-susceptible *S. aureus* (MSSA) in Nigeria (Shittu et al., 2011, 2012; Kolawole et al., 2013), we studied MSSA isolates obtained from various clinical sources in Nigeria using this comprehensive, array-based approach to provide an insight on the major factors associated with infection and disease.

## Materials and methods

### Identification and antibiotic susceptibility testing of *S. aureus* isolates

The isolates (*n* = 52) were obtained from samples processed as part of surveillance activities in the microbiology laboratories of six health care institutions located in Ado-Ekiti, Ile-Ife, Osogbo, Lagos, and Ibadan in South-West Nigeria, and Maiduguri in North-East Nigeria. The duration of collection of isolates was from March 2009 to April, 2010. Only the isolates were analyzed in this study. Preliminary verification as *S. aureus* was based on colony characteristics on blood agar, positive results for catalase, coagulase and DNase tests. Twelve isolates from a previous study (Shittu et al., 2011) were also included in this investigation. Identification was confirmed by Matrix-Assisted Laser Desorption/Ionization-Time Of Flight analysis (MALDI-TOF). Susceptibility testing to penicillin (10 units), cefoxitin (30 μg), doxycycline (30 μg), erythromycin (15 μg), clindamycin (2 μg), gentamicin (10 μg), chloramphenicol (30 μg), and trimethoprim-sulfamethoxazole (1.25/23.75 μg) were determined using the disk diffusion method according to the Clinical Laboratory Standards Institute guidelines (Clinical and Laboratory Standards Institute (CLSI), 2009).

### DNA extraction

*S. aureus* genomic DNA was extracted from an 18–24 h old culture on sheep blood agar using lysis buffer and lysis enhancer (StaphyType Kit, Alere Technologies GmbH, Jena, Germany) and processed using a DNeasy tissue kit (Qiagen, Hilden, Germany).

### Molecular typing of the isolates

Typing of *S. aureus* was based on sequencing of the hypervariable region of the protein A gene (*spa*). The *spa* types were determined using the Ridom StaphType software (Ridom GmbH, Würzburg, Germany, version 2.1.1) (Harmsen et al., 2003). Multilocus sequence typing (MLST) was performed for one isolate of each *spa* type (Enright et al., 2000), as a *spa* type usually belongs to one sequence type (ST) with few exceptions due to homoplasies (Basset et al., 2009, 2012). The allelic profiles and STs were assigned using the MLST *S. aureus* database (www.mlst.net), and the sequence types of the remaining isolates were inferred from the derived MLST data.

### DNA microarray hybridization

The DNA microarray of the StaphyType™ kit (Alere Technologies GmbH, Jena, Germany) was used in this study according to previously established protocols (Monecke et al., 2008). The isolates were grouped with various clonal complexes (CCs) by the imaging software Iconoclust based on comparison of hybridization profiles to a collection of reference strains previously characterized by MLST.

### Splits graph construction

The SplitsTree algorithm (Huson and Bryant, 2006) and software was used to analyze the similarities between hybridization patterns, and network tree construction was performed using SplitsTree 4.10 on default settings (characters transformation, uncorrected P; distance transformation, Neighbor-Net; and variance, ordinary least squares).

## Results

### Identification of *S. aureus* isolates

A total of 52 MSSA (3 and 49 isolates from nasal and clinical sources, respectively) were analyzed (Table 1). The clinical isolates were obtained from wounds and associated infections (*n* = 29; 59.2%), urinary tract infections (*n* = 6; 12.2%), semen/infertility diagnosis (*n* = 4; 8.2%), ocular infections (*n* = 3; 6.1%), and pneumonia (*n* = 2; 4.1%). One isolate each was from otitis media, and blood related infections, while information on three isolates was not available. The clinical isolates were obtained from health care institutions located in Ile-Ife (*n* = 26; 53.1%), Osogbo (*n* = 11; 22.4%), Maiduguri (*n* = 5; 10.2%), Lagos (*n* = 4; 8.2%), Ibadan, and Ado-Ekiti (*n* = 2 isolates each: 4.1%).

**Table 1:** **Characterization of the methicillin-susceptible *S. aureus* (MSSA) from Nigeria based on antibiotyping, microarray analysis, *spa* typing, and MLST**.

**Isolate Number**	**Location**	**Sample/Clinical diagnosis**	**Antibiogram**	**Score (%) (Alere)**	***agr*/Clonal complex (Alere)**	***spa* type**	**MLST**
11486_24	Ile-Ife	Wound Infection	PEN	93.8	agr_III/CC1	t127^¶^	ST1
AB5_28	Osogbo	UTI	PEN, ERY(i)	92.8	agr_III/CC1	t127	ST1
Aro_29	Osogbo	Semen	PEN	94.3	agr_III/CC1	t127	ST1
MD16_4	Not available	Not available	PEN	94.3	agr_III/CC1	t127	ST1
MD20_8^*^	Maiduguri	Wound infection	PEN, ERY(i), CC(i)	93.5	agr_III/CC1	t321^¶^	ST1
6056_34	Osogbo	Urine	PEN	93.9	agr_III/CC1	t10433^¶^	ST1
5675_6	Ile-Ife	Abscess	PEN	91.8	agr_II/CC5	t311	ST5
5221_7	Ile-Ife	Urine	PEN, ERY(i), SXT(i)	93.8	agr_II/CC5	t311	ST5
D23_15	Ile-Ife	Pneumonia	PEN	92.8	agr_II/CC5	t311	ST5
D42_17	Ile-Ife	Adenocarcinoma	PEN, ERY(i)	92.4	agr_II/CC5	t311	ST5
D46_18	Ile-Ife	Wound Infection	PEN, ERY(i)	92.2	agr_II/CC5	t311^¶^	ST5
1423_36	Osogbo	Urine	PEN, ERY(i)	93.8	agr_II/CC5	t442^¶^	ST5
D19_14	Ile-Ife	Not available	PEN	93.5	agr_II/CC5	t688^¶^	ST5
Asu29_27	Osogbo	Otitis media	PEN, DO, ERY(i)	91.9	agr_II/CC5	t1277^¶^	ST5
3211_30	Osogbo	Wound Infection	PEN	92.9	agr_II/CC5	t3235^¶^	ST5
6773_11	Ile-Ife	Wound Infection	PEN	93.6	agr_I/CC7	t091^¶^	ST789
N37_19	Ile-Ife	Erythematous lesion	PEN, SXT	90	agr_I/CC8	t064^¶^	ST2427
UC45_37	Ibadan	Eye swab	PEN, GM, CHL, SXT	91.3	agr_I/CC8	t2658^¶^	ST2427
55_40	Ado-Ekiti	Wound Infection	PEN, DO(i), GM, CHL, SXT	90.3	agr_I/CC8	t2658	ST2427
OS39_13^*^	Lagos	Semen/Infertility	PEN, DO(i), SXT	91.7	agr_I/CC8	t951^¶^	ST8
11450_23	Ile-Ife	Sputum	PEN	92.9	agr_II/CC15	t084	ST15
5189_1	Ile-Ife	Advanced Cancer	PEN	94	agr_II/CC15	t084	ST15
189_2	Ile-Ife	Blood	PEN, DO(i), ERY(i)	93.9	agr_II/CC15	t084^¶^	ST15
4013_14^*^	Ile-Ife	Wound infection	PEN	94.9	agr_II/CC15	t084	ST15
5828_5	Ile-Ife	Abscess	susceptible to all antibiotics tested	94.4	agr_II/CC15	t2216^¶^	ST15
MD7_3^*^	Maiduguri	Semen/Infertility	PEN, ERY(i)	94.6	agr_II/CC15	t2216	ST15
MD19_11^*^	Maiduguri	Wound infection	PEN	94.4	agr_II/CC15	t2216	ST15
S13_6^*^	Lagos	Urinary Tract Infection	PEN, ERY(i), SXT	93.1	agr_I/CC25	t3772^¶^	ST25
3925_32	Osogbo	Wound Infection	PEN, ERY(i), SXT	91.4	agr_I/CC25	t10183^¶^	ST25
6073_3	Not available	Not available	PEN, DO	91.7	agr_III/CC30	t017^¶^	ST30
D30_16	Ile-Ife	Cholecystitis	PEN	94.7	agr_III/CC30	t318^¶^	ST30
6506_2	Osogbo	Wound Infection	PEN, ERY(i), CC(i)	91.4	agr_III/CC30	t318	ST30
NS7708_22	Ile-Ife	Nasal swab/screening	PEN, ERY(i)	94.7	agr_III/CC30	t318	ST30
54_39	Ado-Ekiti	Wound Infection	PEN	94.4	agr_III/CC30	t318	ST30
S12_7^*^	Lagos	Wound infection	PEN, ERY(i)	93.8	agr_III/CC30	t318	ST30
OS41_10^*^	Lagos	Wound infection	PEN	93.1	agr_III/CC30	t318	ST30
6330_4	Ile-Ife	Osteomyelitis	PEN	94.3	agr_III/CC30	t318	ST30
NS2907_21	Ile-Ife	Nasal swab/screening	PEN, ERY(i), CC(i)	91.8	agr_I/CC45	t095^¶^	ST508
3950_33	Osogbo	Urine	PEN	91.5	agr_I/CC45	t10434^¶^	ST508
GDC_35	Osogbo	Semen	PEN	94.9	agr_III/CC80	t934^¶^	ST80
MD14_2^*^	Maiduguri	Wound infection	PEN, DO(i)	92.9	agr_I/CC97	t458^¶^	ST97
ZU_26	Ile-Ife	Unavailable	PEN, ERY(i)	89.3	agr_IV/CC121	t159^¶^	ST121
UC47_38	Ibadan	Eye swab	PEN, DO, ERY(i), CC(i)	92.1	agr_IV/CC121	t159	ST121
W10_5^*^	Ile-Ife	Wound infection	PEN, ERY(i)	91.8	agr_IV/CC121	t314^¶^	ST121
MD_9^*^	Maiduguri	Wound infection	PEN, ERY(i), CC(i)	92.1	agr_IV/CC121	t314	ST121
6376_3	Ile-Ife	Abscess	PEN, DO(i)	93.1	agr_IV/CC121	t2304^¶^	ST121
6540_10	Ile-Ife	Bone Marrow Infection	PEN	93.5	agr_IV/CC121	t2304	ST121
NS2986_20	Ile-Ife	Nasal swab/screening	PEN, DO, ERY(i), CC(i), SXT(i)	92.8	agr_IV/CC121	t2304	ST121
3920_31	Osogbo	Aspirate	PEN	92.8	agr_IV/CC121	t2304	ST121
D3_12	Ile-Ife	Cervical cancer	PEN, ERY(i)	94.6	agr_I/ST152	t355^¶^	ST152
D12_13	Ile-Ife	Ocular infection	PEN	94.6	agr_I/ST152	t355	ST152
W7.2_4^*^	Ile-Ife	Wound infection	PEN	96.3	agr_I/ST152	t355	ST152

* S. aureus analyzed in a previous study; (i): intermediate susceptibility; agr, accessory gene regulator; PEN, Penicillin; DO, Doxycycline; ERY, Erythromycin; CLI, Clindamycin; GEN, Gentamicin; CHL, Chloramphenicol; SXT, trimethoprim/sulfamethoxazole; CC, Clonal Complex; ST, Sequence type.

¶ spa types selected for Multilocus sequence typing (MLST); Sequence types (STs) of the remaining isolates were inferred from the derived MLST data.

### Antibiotic susceptibility testing

All the isolates were susceptible to cefoxitin and 98.1% (*n* = 51) were resistant to penicillin. Only two isolates each exhibited resistance to chloramphenicol and gentamicin, and four to doxycycline. Intermediate susceptibility to clindamycin and erythromycin were identified in six and 21 isolates, respectively. The predominant antibiotype was resistance only to penicillin (*n* = 23; 44.2%), and resistance to penicillin with intermediate susceptibility to erythromycin (*n* = 10; 19.2%) (Table 1).

### Sequence based typing (*spa* and MLST)

A total of 26 *spa* types were identified among the 52 MSSA isolates and the most common were t318 (*n* = 7), t311 (*n* = 5), t084, t127, and t2304 (*n* = 4 each). Based on MLST, the MSSA were classified into 13 sequence types (STs) (Table 1).

### DNA microarray analysis

The assay confirmed the identity of the isolates (*S. aureus*) by positive results for specific markers including rndD1 (domain 1 of 23S rRNA), protein A (*spa*), glyceraldehyde 3-phosphate dehydrogenase (*gapA*), catalase A (*katA*), thermostable nuclease (*nuc*), and staphylococcal accessory regulator A (*sarA*) (Supplementary Materials 1, 2). The hybridization profiles revealed that the 52 MSSA isolates clustered in 12 different CCs. More than half (55.8%) of the CCs were associated with the genetic background common to the major methicillin-resistant *S. aureus* (MRSA) clones i.e., CC1 (*n* = 6 isolates), CC5 (*n* = 9), CC8 (*n* = 4), CC30 (*n* = 8), and CC45 (*n* = 2). The rest were assigned with CC7 (*n* = 1), CC15 (*n* = 7), CC25 (*n* = 2), CC80 (*n* = 1), CC97 (*n* = 1), CC121 (*n* = 8), and CC152 (*n* = 3).

### Antibiotic resistance genes

A total of 69.2% (*n* = 36) of the isolates yielded a hybridization signal for the beta-lactamase gene (*blaZ*) and only 10 and three isolates were positive for the tetracycline resistance genes (*tetK* and *tetM*), respectively. The two MSSA in CC8 which exhibited phenotypic resistance to chloramphenicol and gentamicin possessed the corresponding resistance genes (*cat* and *aacA-aphD*). In addition, the single CC80 isolate was positive for the lincosamide resistance gene (*lnuA*).

### Accessory gene regulator and capsular typing

**Figure 1:**
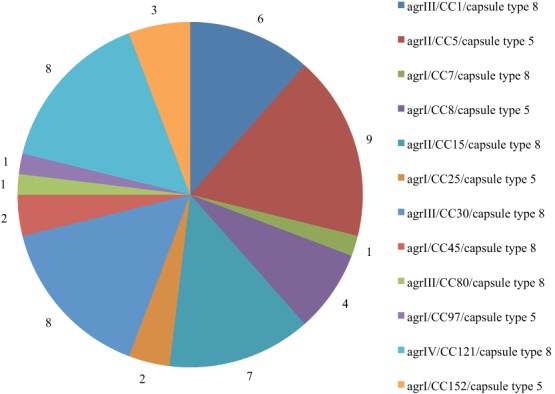
**Distribution of *agr*/CCs/capsule type of MSSA in Nigeria**.

The distribution of *agr*/CCs/capsule types for the MSSA is indicated in Figure 1. Overall, 13 (25%) isolates assigned to different clonal lineages (CC7, CC8, CC25, CC45, CC97, and CC152) were associated with *agr* group I, 16 (30.2%; CC5 and CC15) with group II, and 15 (28.8%; CC1, CC30 and CC80) with group III. CC121 was the only representative for *agr* group IV (*n* = 8; 15.4%) (Table 1). The capsule type 8 was the most frequent and detected in 33 (63.5%) isolates affiliated with CC1, CC7, CC15, CC30, CC45, CC80, and CC121. The remaining isolates (20; 38.5%) belonged to capsule type 5 (assigned with CC5, CC8, CC25, CC97, and CC152).

### Enterotoxin genes

PVL-positive isolates (*n* = 27) belonged to CC1, CC5, CC15, CC30, CC80, CC121, and CC152 (Supplementary Material 1). Moreover, the *lukF* gene (haemolysin gamma; component B) was universally detected in all the CCs and the *lukE* genes was a common feature except with MSSA isolates in CC30, CC45, and CC152 (Supplementary Material 3). With respect to the carriage of superantigen genes, only three MSSA (one isolate in CC1 and two in CC45) tested positive for the toxic shock syndrome toxin gene (*tst-1*) (Supplementary Material 1). All the isolates in this study lacked a hybridization signal for the enterotoxin E gene (see Supplementary Material 2) and the enterotoxin genes were not detected in MSSA assigned with CC80, CC97, and CC152. In the haemolysin gene family, almost all (98.1%) the isolates in the various CCs possessed the haemolysin alpha and delta genes (*hla, hld*), while the haemolysin beta gene (*hlb*) was identified in the various CCs except in CC15, CC45, and CC152.

### Microbial surface components recognizing adhesive matrix molecule (MSCRAMM) genes

All the isolates were negative for the surface protein involved in biofilm production (*bap*), but possessed the genes for the inter-cellular adhesion protein (*icaA/C/D*) (CC152 isolates were *icaC* negative). The genes for clumping factor A (*clfA*), cell surface elastin binding protein (*ebpS*), fibronectin-binding protein A (*fnbA*) and proteases (*sspA, sspB*, and *sspP*) were detected in all the isolates (Supplementary Materials 1, 3).

### Splits tree analysis

**Figure 2:**
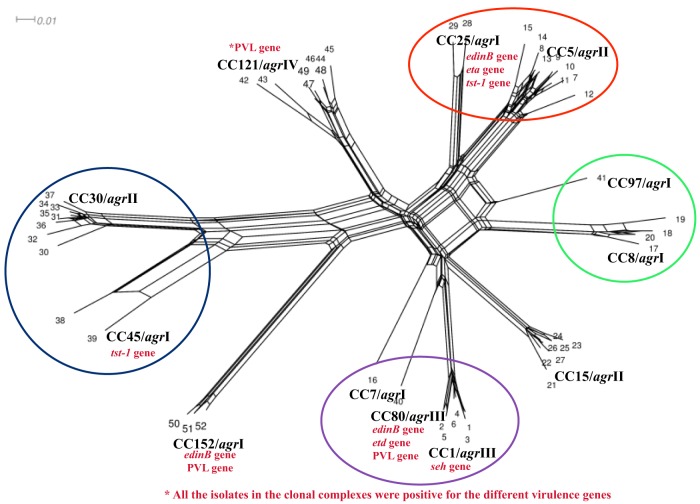
**Splits tree graph based on hybridization profile of the MSSA isolates**. The results of all array hybridization experiments were arranged in a matrix. Columns represent the target genes and the rows represent the number of experiments; 1, positive; 0, negative; −, ambiguous. Converted to “sequences”: 1, c; 0, g; −, c.

The analysis identified four main clusters (CC5/CC25; CC8/CC97; CC1/CC7/CC80, and CC30/CC45) indicating the phylogenetic relationship between the isolates (Figure 2).

## Discussion

We observed a complete agreement between DNA microarray analysis and MLST in the delineation of the isolates (Table 1), showing that the hybridization profile could be used to predict the lineages. Furthermore, the heterogeneous and divergent nature of the isolates observed in this study provided evidence on the overall higher diversity of MSSA compared with MRSA (Deurenberg and Stobberingh, 2008; Goering et al., 2008; Ghasemzadeh-Moghaddam et al., 2011; Ruffing et al., 2012; Blomfeldt et al., 2013; Rasmussen et al., 2013, 2014). In Nigeria, many diagnostic microbiology laboratories rely on the disc diffusion technique for antibiotic susceptibility testing, but this protocol does not provide information on the nature of resistance genes. The antibiotic susceptibility results observed in this study were in accordance with the corresponding resistance gene profiles by DNA microarray. MSSA isolates that exhibited full resistance to trimethoprim-sulfamethoxazole clustered with CC8 and CC25, but were *dfrS1* negative indicating that a different mechanism could be attributed to resistance. A recent study (Nurjadi et al., 2014) has provided strong evidence that the *dfrG* gene is the predominant trimethoprim resistance determinant on *S. aureus* in Africa. Overall, resistant determinants for antibiotics, heavy metal and quaternary ammonium compounds were observed more often in CC8 than other CCs (Supplementary Materials 1, 3).

The accessory gene regulator (*agr*) and capsule typing methods are useful front-line tools for the characterization of *S. aureus* (Goerke et al., 2005). Hybridization signals for *agr* type I and IV were observed for one, three, and four isolates grouped with CC25, CC152, and CC121, respectively (Supplementary Materials 1, 2). This could be attributed to possible cross-hybridization as the alleles for the two *agr* types are closely related (Monecke et al., 2010). Our observations on CCs and *agr* groups were similar to previous reports on MSSA in five major African towns (Breurec et al., 2010), Gabon (Ateba Ngoa et al., 2012), and Nigeria (Ghebremedhin et al., 2009; Kolawole et al., 2013). In addition, our study also support the view (Wright et al., 2005; Holtfreter et al., 2007; Rasmussen et al., 2014) that an *agr* type may be detected in isolates which are assigned to genetically diverse CCs, whereas, it is also associated with specific CCs. The dominance of capsule type 8 in MSSA is consistent with data from Gabon (Schaumburg et al., 2011), Norway (Blomfeldt et al., 2013), and Sweden (Rasmussen et al., 2013, 2014).

Staphylococcal enterotoxins are typically encoded by genes located on mobile genetic elements (Baba et al., 2002). The *egc* cluster (*seg*+*sei*+*sem*+*sen*+*seo*+*seu*) is located on the genomic island vSAβ and reported to be associated with specific clonal types regardless of the geographical strain distribution (Lindsay and Holden, 2006). In this investigation, the *egc*-enterotoxin gene cluster was a unique feature for CC5, CC25, CC30, CC45, and CC121. Previous studies have indicated that the cluster is predominantly present in MSSA assigned with CC5, CC25, CC30, and CC45 (Van Trijp et al., 2010; Rasmussen et al., 2013). The *seh* gene is linked to the staphylococcal cassette chromosome *mec* (SCC*mec* elements) and reported to be restricted to the CC1 genomic background (Baba et al., 2002). Moreover, the *seh* gene has also been reported mainly in MSSA-CC30 (Blomfeldt et al., 2013). Nevertheless, our observation on *seh*-positive MSSA-CC1 is in agreement with previous reports (Chen et al., 2013; Rasmussen et al., 2013).

The genes associated with staphylococcal complement inhibitor (*scn*) and staphylokinase (*sak*) were also widely distributed across the CCs but CC15 isolates were *sak* gene negative. Virulence associated with the exfoliative toxins has been identified to cause epidermal cleavage in staphylococcal scalded skin syndrome (SSSS) and bullous impetigo (Ladhani et al., 1999). The exfoliative toxin D (ETD) is a 27-kDa protein which causes epidermal blisters in newborn mice (Yamasaki et al., 2006). The epidermal cell differentiation factors (EDIN) target and inhibit the small host protein RhoA, a master regulator of the host cell actin cytoskeleton (Inoue et al., 1991; Jaffe and Hall, 2005; Aktories, 2011). Furthermore, the edin-isoform (*edinB*) and *etd* genes are located in tandem in a *S. aureus etd* pathogenicity island in a chromosome of *etd*-positive *S. aureus* strains (Yamaguchi et al., 2002). A strong association of the *etd* gene with invasive CC25 *S. aureus* isolates has also been reported. In this study, all the isolates assigned with CC25 and CC80 were *etd*-positive, which is in agreement with a previous study in Nigeria (Shittu et al., 2011). Moreover, MSSA grouped with CC25, CC80, and CC152 were *edinB* positive but CC152 isolates were *etd* negative. Our observations were similar to a study on the distribution of the *edin* gene in *S. aureus* from diabetic foot ulcers (Messad et al., 2013). A study in MSSA bacteremia isolates in Sweden showed that the collagen binding protein (Cna) was detected in CC1, CC30, and CC45. Our report identified the gene in isolates assigned with CC1, CC30, CC45, CC121, and CC152.

Our study has a number of limitations. Although all isolates were of human origin, and the large majority was obtained from clinical samples, a clear distinction between commensal and clinical strains could not be made based on the available information. An association of isolates within the context of endemicity i.e., nosocomial vs. community associated infections, is also not clear. Furthermore, whereas the microarray analytical database is exhaustive, well-characterized, and validated with isolates from all continents, the attribution of CCs is based on the hybridization reactions and resulting microarray profile rather than gene sequencing, and a positive signal does not necessarily imply the presence of gene product (e.g., protein). In addition, the microarray method was unable to separate ST8 from ST2427. This might be due to the close phylogenetic relation of both STs as they are single locus variants (ST8: 3−3−1−1−4−4−3 and ST2427: 3−3−297−1−4−4−3). Finally, with a collection of 52 isolates studied, and a large number of genes and genetic profile ascertained by microarray, the potential for individual statistical comparisons is limited. Yet, with this comprehensive genetic-analytical approach performed on a clinical isolate collection obtained from patients of various medical institutions in a sub-Saharan African country, Nigeria, a number of important observations could be made which clearly characterize and demarcate the clonal distribution as well as the virulence gene equipment.

More than one half (55.8%; *n* = 29) of these MSSA isolates were associated with a genetic background which is attributable to classic methicillin-resistant *S. aureus* (MRSA) clones. PVL-positive isolates were identified in seven of the 12 CCs. Moreover, toxin genes were observed to be distributed mainly with certain clonal types, and in agreement with previous investigations (Holtfreter et al., 2007; Monecke et al., 2008). Antibiotic resistance gene profiles of the isolates by the DNA microarray demonstrated concordant results with data on antibiotic susceptibility testing. The array-based, comprehensive approach has been shown to yield such diverse CC and gene specific results on an isolate collection from sub-Saharan Africa. Overall, microarray analysis proved to be a useful tool to provide useful information on antibiotic resistance, population structure and various virulence factor profiles associated with infection and disease. It is assumed that these findings might be useful for a better understanding of clinical staphylococcal disease presentation, patient care and for assistance in outbreak investigation in health care institutions in a country such as Nigeria. Moreover, our study also underlines the need for further trials employing well-controlled, prospectively collected clinical isolates to delineate the genetic pathogen profile in conjunction with the clinical disease presentation in sub-Saharan Africa.

## Author contributions

AS, UR, GP, FS, LM, and MH conceived the study, OO, KO, AR conducted the sample collection and preliminary identification of the isolates. AS performed the microarray technique, AS and UR analyzed the microarray data, and AS wrote the manuscript (with input from all authors). All authors read and approved the final version of the manuscript.

## Funding

The stay of AS at the Institute of Medical Microbiology and Hygiene, Institute of Medical Microbiology, Saarland University Medical Centre, Homburg/Saar and the University Hospital Münster, Münster, Germany was supported by the Third World Academy of Science and Deutsche Forschungsgemeinschaft (TWAS-DFG award) and the Deutsche Forschungsgemeinschaft (PAK296, Ei 247/8, He 1850/9−1, and He 1850/11−1).

### Conflict of interest statement

The authors declare that the research was conducted in the absence of any commercial or financial relationships that could be construed as a potential conflict of interest.

## Abbreviations

Agr: accessory gene regulator
CC: Clonal complex
CLSI: Clinical Laboratory Standards Institute
MSSA: Methicillin susceptible *Staphylococcus aureus*
MLST: Multilocus sequence typing
PVL: Panton-Valentine Leukocidin
*S*.aureus: *Staphylococcus aureus*
SCC*mec*: Staphylococcal chromosome cassette *mec*
*spa*: *Staphylococcus aureus* protein A
ST: Sequence Type.
