# Supplementary material for: An assessment on DNA microarray and sequence-based methods for the characterization of methicillin-susceptible Staphylococcus aureus from Nigeria
Source: Front Microbiol. 2015 Oct 20;6:1160. doi: 10.3389/fmicb.2015.01160 (PMC4612102; doi:10.3389/fmicb.2015.01160)
Supplement: Supplementary file 3 [file DataSheet3.DOC]

Supplementary Material 3: Distribution (in percentage) of some selected genes among methicillin-susceptible *Staphylococcus aureus* isolates from Nigeria

| **Category** | **Gene** | **Description** | **CC1 (n=6)**  **%** | **CC5 (n=9)**  **%** | **CC7 (n=1)**  **%** | **CC8 (n=4)**  **%** | **CC15 (n=7)**  **%** | **CC25 (n=2)**  **%** | **CC30 (n=8)**  **%** | **CC45**  **(n=2)**  **%** | **CC80**  **(n=1)**  **%** | **CC97 (n=1)**  **%** | **CC121 (n=8)**  **%** | **CC152**  **(n=3)**  **%** |
| --- | --- | --- | --- | --- | --- | --- | --- | --- | --- | --- | --- | --- | --- | --- |
| RESISTANCE : PENICILLINASE | *blaZ* | beta-lactamase | 100.0 | 22.2 | 100.0 | 100.0 | 71.4 | 100.0 | 62.5 | 50..0 | 100.0 | 100.0 | 87.5 | 33.3 |
|  | *blaI* | beta-lactamase repressor (inhibitor) | 83.3 | 22.2 | 100.0 | 100.0 | 71.4 | 100.0 | 62.5 | 50.0 | 100.0 | 100.0 | 87.5 | 33.3 |
|  | *blaR* | beta-lactamase operon regulatory protein | 100.0 | 22.2 | 100.0 | 100.0 | 71.4 | 100.0 | 62.5 | 50.0 | 100.0 | 100.0 | 87.5 | 33.3 |
| RESISTANCE : MACROLIDE-LINCOSAMIDE-STREPTOGRAMIN B ANTIBIOTICS | *ermA* | macrolide/clindamycin resistance | 0.0 | 0.0 | 0.0 | 0.0 | 0.0 | 0.0 | 0.0 | 0.0 | 0.0 | 0.0 | 0.0 | 0.0 |
|  | *ermB* | macrolide/clindamycin resistance | 0.0 | 0.0 | 0.0 | 0.0 | 0.0 | 0.0 | 0.0 | 0.0 | 0.0 | 0.0 | 0.0 | 0.0 |
|  | *ermC* | macrolide/clindamycin resistance | 0.0 | 0.0 | 0.0 | 0.0 | 0.0 | 0.0 | 0.0 | 0.0 | 0.0 | 0.0 | 0.0 | 0.0 |
|  | *lnu(A)* | lincosamide resistance gene | 0.0 | 0.0 | 0.0 | 0.0 | 0.0 | 0.0 | 0.0 | 0.0 | 100.0 | 0.0 | 0.0 | 0.0 |
|  | *msr(A)* | macrolide resistance gene | 0.0 | 0.0 | 0.0 | 0.0 | 0.0 | 0.0 | 0.0 | 0.0 | 0.0 | 0.0 | 0.0 | 0.0 |
|  | *mef(A)* | macrolide resistance gene | 0.0 | 0.0 | 0.0 | 0.0 | 0.0 | 0.0 | 0.0 | 0.0 | 0.0 | 0.0 | 0.0 | 0.0 |
|  | *mph(C)* | macrolide resistance gene | 0.0 | 0.0 | 0.0 | 0.0 | 0.0 | 0.0 | 0.0 | 0.0 | 0.0 | 0.0 | 0.0 | 0.0 |
|  | *vat(A)* | virginiamycin-A-resistance gene | 0.0 | 0.0 | 0.0 | 0.0 | 0.0 | 0.0 | 0.0 | 0.0 | 0.0 | 0.0 | 0.0 | 0.0 |
|  | *vat(B)* | streptogramin-A-resistance gene | 0.0 | 0.0 | 0.0 | 0.0 | 0.0 | 0.0 | 0.0 | 0.0 | 0.0 | 0.0 | 0.0 | 0.0 |
|  | *vga(A)* | resistance gene to streptogramin A, lincosamide, pleuromutilin | 0.0 | 0.0 | 0.0 | 0.0 | 0.0 | 0.0 | 0.0 | 0.0 | 0.0 | 0.0 | 0.0 | 0.0 |
|  | *vga(A) (BM 3327)* | resistance gene to streptogramin A (allele from strain BM 3327) | 0.0 | 0.0 | 0.0 | 0.0 | 0.0 | 0.0 | 0.0 | 0.0 | 0.0 | 0.0 | 0.0 | 0.0 |
|  | *vgb(A)* | virginiamycin-B/pristinamycin resistance gene | 0.0 | 0.0 | 0.0 | 0.0 | 0.0 | 0.0 | 0.0 | 0.0 | 0.0 | 0.0 | 0.0 | 0.0 |
| RESISTANCE : AMINOGLYCOSIDES | *aacA-aphD* | gentamicin/tobramycin resistance gene | 0.0 | 0.0 | 0.0 | 50.0 | 0.0 | 0.0 | 0.0 | 0.0 | 0.0 | 0.0 | 0.0 | 0.0 |
|  | *aadD* | tobramycin resistance gene | 0.0 | 0.0 | 0.0 | 0.0 | 0.0 | 0.0 | 0.0 | 0.0 | 0.0 | 0.0 | 0.0 | 0.0 |
|  | *aphA3* | neo-/kanamycin resistance gene | 0.0 | 0.0 | 100.0 | 0.0 | 0.0 | 0.0 | 0.0 | 0.0 | 0.0 | 0.0 | 0.0 | 0.0 |
| RESISTANCE : MISCELLANEOUS GENES | *sat* | streptothricin resistance gene | 0.0 | 0.0 | 100.0 | 0.0 | 0.0 | 0.0 | 0.0 | 0.0 | 0.0 | 0.0 | 0.0 | 0.0 |
|  | *dfrS1* | trimethoprim resistance gene | 0.0 | 0.0 | 0.0 | 0.0 | 0.0 | 0.0 | 0.0 | 0.0 | 0.0 | 0.0 | 0.0 | 0.0 |
|  | *far1* | fusidic acid resistance gene | 0.0 | 0.0 | 0.0 | 0.0 | 0.0 | 0.0 | 0.0 | 0.0 | 0.0 | 0.0 | 0.0 | 0.0 |
|  | *Q6GD50* | hypothetical Protein associated with fusidic acid resistance | 0.0 | 0.0 | 0.0 | 0.0 | 0.0 | 0.0 | 0.0 | 0.0 | 0.0 | 0.0 | 0.0 | 0.0 |
|  | *mupA* | mupirocin resistance protein | 0.0 | 0.0 | 0.0 | 0.0 | 0.0 | 0.0 | 0.0 | 0.0 | 0.0 | 0.0 | 0.0 | 0.0 |
|  | *tet(K)* | tetracycline resistance gene | 0.0 | 0.0 | 100.0 | 75.0 | 0.0 | 0.0 | 0.0 | 0.0 | 100.0 | 100.0 | 50.0 | 0.0 |
|  | *tet(M)* | tetracycline resistance gene | 0.0 | 11.1 | 0.0 | 0.0 | 14.3 | 0.0 | 12.5 | 0.0 | 0.0 | 0.0 | 0.0 | 0.0 |
|  | *Cat* | chloramphenicol resistance gene | 0.0 | 0.0 | 0.0 | 50.0 | 0.0 | 0.0 | 0.0 | 0.0 | 0.0 | 0.0 | 0.0 | 0.0 |
|  | *Cfr* | 23S rRNA methyltransferase, resistance gene to phenicols, lincosamides | 0.0 | 0.0 | 0.0 | 0.0 | 0.0 | 0.0 | 0.0 | 0.0 | 0.0 | 0.0 | 0.0 | 0.0 |
|  | *fexA* | chloramphenicol/florfenicol resistance gene | 0.0 | 0.0 | 0.0 | 0.0 | 0.0 | 0.0 | 0.0 | 0.0 | 0.0 | 0.0 | 0.0 | 0.0 |
|  | *fosB* | metallothiol transferase | 0.0 | 100.0 | 0.0 | 100.0 | 100.0 | 100.0 | 100.0 | 0.0 | 0.0 | 0.0 | 100.0 | 0.0 |
| RESISTANCE : EFFLUX SYSTEMS | *qacA* | quaternary ammonium compound resistance gene protein A | 0.0 | 0.0 | 0.0 | 0.0 | 0.0 | 0.0 | 0.0 | 0.0 | 0.0 | 0.0 | 0.0 | 0.0 |
|  | *qacC* | quaternary ammonium compound resistance gene protein C | 0.0 | 0.0 | 0.0 | 50.0 | 0.0 | 0.0 | 0.0 | 0.0 | 0.0 | 0.0 | 0.0 | 0.0 |
|  | *tetEfflux* | putative transport protein (tetEfflux) | 100.0 | 100.0 | 100.0 | 100.0 | 100.0 | 100.0 | 100.0 | 100.0 | 100.0 | 100.0 | 100.0 | 100.0 |

| **Category** | **Gene** | **Description** | **CC1 (n=6)**  **%** | **CC5 (n=9)**  **%** | **CC7 (n=1)**  **%** | **CC8 (n=4)**  **%** | **CC15 (n=7)**  **%** | **CC25 (n=2)**  **%** | **CC30 (n=8)**  **%** | **CC45**  **(n=2)**  **%** | **CC80**  **(n=1)**  **%** | **CC97 (n=1)**  **%** | **CC121 (n=8)**  **%** | **CC152**  **(n=3)**  **%** |
| --- | --- | --- | --- | --- | --- | --- | --- | --- | --- | --- | --- | --- | --- | --- |
| RESISTANCE : GLYCOPEPETIDES | *vanA* | vancomycin resistance gene | 0.0 | 0.0 | 0.0 | 0.0 | 0.0 | 0.0 | 0.0 | 0.0 | 0.0 | 0.0 | 0.0 | 0.0 |
|  | *vanB* | vancomycin resistance gene from enterococci and clostridium | 0.0 | 0.0 | 0.0 | 0.0 | 0.0 | 0.0 | 0.0 | 0.0 | 0.0 | 0.0 | 0.0 | 0.0 |
|  | *vanZ* | teicoplanin resistance gene from enterococci | 0.0 | 0.0 | 0.0 | 0.0 | 0.0 | 0.0 | 0.0 | 0.0 | 0.0 | 0.0 | 0.0 | 0.0 |
| VIRULENCE : TOXIC SHOCK.TOXIN | *tst1* | toxic shock syndrome toxin | 16.7 | 0.0 | 0.0 | 0.0 | 0.0 | 0.0 | 0.0 | 100.0 | 0.0 | 0.0 | 0.0 | 0.0 |
| VIRULENCE : ENTEROTOXINS  VIRULENCE : ENTEROTOXINS | *sea* | enterotoxin A | 66.7 | 33.3 | 100.0 | 75.0 | 14..3 | 0.0 | 12.5 | 0.0 | 0.0 | 0.0 | 0.0 | 0.0 |
|  | *seb* | enterotoxin B | 0.0 | 11.1 | 0.0 | 100.0 | 0.0 | 0.0 | 0.0 | 0.0 | 0.0 | 0.0 | 75.0 | 0.0 |
|  | *sec* | enterotoxin C | 0.0 | 0.0 | 0.0 | 0.0 | 0.0 | 100.0 | 12.5 | 100.0 | 0.0 | 0.0 | 0.0 | 0.0 |
|  | *sed* | enterotoxin D | 0.0 | 11.1 | 0.0 | 0.0 | 0.0 | 0.0 | 0.0 | 0.0 | 0.0 | 0.0 | 0.0 | 0.0 |
|  | *see* | enterotoxin E | 0.0 | 0.0 | 0.0 | 0.0 | 0.0 | 0.0 | 0.0 | 0.0 | 0.0 | 0.0 | 0.0 | 0.0 |
|  | *seh* | enterotoxin H | 100.0 | 0.0 | 0.0 | 0.0 | 0.0 | 0.0 | 0.0 | 0.0 | 0.0 | 0.0 | 0.0 | 0.0 |
|  | *seg/i/m/n/o/u* | egc-locus, comprising entg/i/m/n/o/u | 0.0 | 100.0 | 0.0 | 0.0 | 0.0 | 100.0 | 100.0 | 100.0 | 0.0 | 0.0 | 100.0 | 0.0 |
|  | *sei*  *sek* | enterotoxin I  enterotoxin K | 0.0  100.0 | 100.0  0.0 | 0.0  0.0 | 0.0  100.0 | 0.0  0.0 | 100.0  0.0 | 100.0  0.0 | 100.0  0.0 | 0.0  0.0 | 0.0  0.0 | 75.0  12.5 | 0.0  0.0 |
|  | *sem* | enterotoxin-like gene/ protein M | 0.0 | 100.0 | 0.0 | 0.0 | 0.0 | 100.0 | 100.0 | 100.0 | 0.0 | 0.0 | 100.0 | 0.0 |
|  | *sen*  *seq* | enterotoxin-like gene/protein N  enterotoxin Q | 0.0  100.0 | 100.0  0.0 | 0.0  0.0 | 0.0  100.0 | 0.0  0.0 | 100.0  0.0 | 100.0  0.0 | 100.0  0.0 | 0.0  0.0 | 0.0  0.0 | 100.0  12.5 | 0.0  0.0 |
| VIRULENCE : LEUKOCIDINS AND  HAEMOLYSINS | *lukF* | haemolysin gamma/leukocidin, component B (F) | 100.0 | 88.9 | 100.0 | 100.0 | 100.0 | 100.0 | 100.0 | 100.0 | 100.0 | 100.0 | 100.0 | 66.7 |
|  | *lukS* | haemolysin gamma/leukocidin, component C (S) | 100.0 | 100.0 | 0.0 | 100.0 | 100.0 | 100.0 | 100.0 | 0.0 | 100.0 | 100.0 | 100.0 | 0.0 |
|  | *hlgA* | haemolysin gamma, component A | 100.0 | 100.0 | 100.0 | 100.0 | 100.0 | 100.0 | 100.0 | 100.0 | 100.0 | 100.0 | 100.0 | 100.0 |
|  | *lukF/S-PV* | Panton Valentine leukocidin | 66.7 | 55.6 | 0.0 | 0.0 | 14.3 | 0.0 | 62.5 | 0.0 | 100.0 | 0.0 | 100.0 | 100.0 |
|  | *lukF-PV (P83)* | F component of leukocidin from ruminants | 0.0 | 0.0 | 0.0 | 0.0 | 0.0 | 0.0 | 0.0 | 0.0 | 0.0 | 0.0 | 0.0 | 0.0 |
|  | *lukD* | Leukocidin D component | 100.0 | 100.0 | 100.0 | 100.0 | 85.7 | 100.0 | 0.0 | 0.0 | 100.0 | 100.0 | 87.5 | 0.0 |
|  | *lukE* | Leukocidin E component | 100.0 | 100.0 | 100.0 | 100.0 | 85.7 | 50.0 | 0.0 | 0.0 | 100.0 | 100.0 | 100.0 | 0.0 |
|  | *hla* | haemolysin alpha | 100.0 | 100.0 | 100.0 | 100.0 | 100.0 | 100.0 | 100.0 | 100.0 | 100.0 | 100.0 | 87.5 | 100.0 |
|  | *hlb*  *hld* | haemolysin beta  haemolysin delta | 100.0  100.0 | 100.0  100.0 | 100.0  100.0 | 100.0  100.0 | 0.0  100.0 | 100.0  100.0 | 100.0  87.5 | 0.0  100.0 | 100.0  100.0 | 100.0  100.0 | 100.0  100.0 | 0.0  100.0 |
| VIRULENCE : GENES ASSOCIATED WITH HEMOLYSIN BETA CONVERTING PHAGES | *sak* | staphylokinase | 100.0 | 100.0 | 100.0 | 75.0 | 0.0 | 100.0 | 100.0 | 100.0 | 100.0 | 100.0 | 100.0 | 100.0 |
|  | *chp* | chemotaxis-inhibiting protein (CHIPS) | 66.7 | 88.9 | 0.0 | 0.0 | 100.0 | 100.0 | 87.5 | 100.0 | 0.0 | 0.0 | 0.0 | 0.0 |
|  | *scn* | staphylococcal complement inhibitor | 100.0 | 100.0 | 100.0 | 50.0 | 100.0 | 100.0 | 100.0 | 100.0 | 100.0 | 100.0 | 87.5 | 100.0 |
| VIRULENCE: EXFOLIATIVE TOXINS | *eta* | exfoliative toxin A | 0.0 | 0.0 | 0.0 | 0.0 | 28.6 | 0.0 | 0.0 | 0.0 | 0.0 | 0.0 | 0.0 | 0.0 |
|  | *etb* | exfoliative toxin B | 0.0 | 0.0 | 0.0 | 0.0 | 0.0 | 0.0 | 0.0 | 0.0 | 0.0 | 0.0 | 0.0 | 0.0 |
|  | *etd* | exfoliative toxin D | 0.0 | 0.0 | 0.0 | 0.0 | 0.0 | 100.0 | 0.0 | 0.0 | 100.0 | 0.0 | 0.0 | 0.0 |

| **Category** | **Gene** | **Description** | **CC1 (n=6)**  **%** | **CC5 (n=9)**  **%** | **CC7 (n=1)**  **%** | **CC8 (n=4)**  **%** | **CC15 (n=7)**  **%** | **CC25 (n=2)**  **%** | **CC30 (n=8)**  **%** | **CC45**  **(n=2)**  **%** | **CC80**  **(n=1)**  **%** | **CC97 (n=1)**  **%** | **CC121 (n=8)**  **%** | **CC152**  **(n=3)**  **%** |
| --- | --- | --- | --- | --- | --- | --- | --- | --- | --- | --- | --- | --- | --- | --- |
| VIRULENCE : EPITHELIAL DIFFERENTIATION INHIBITOR | *edinA* | epidermal cell differentiation inhibitor | 0.0 | 0.0 | 0.0 | 0.0 | 0.0 | 0.0 | 0.0 | 0.0 | 0.0 | 0.0 | 0.0 | 0.0 |
|  | *edinB* | epidermal cell differentiation inhibitor B | 0.0 | 0.0 | 0.0 | 0.0 | 0.0 | 100.0 | 0.0 | 0.0 | 100.0 | 0.0 | 0.0 | 100.0 |
|  | *edinC* | epidermal cell differentiation inhibitor C | 0.0 | 0.0 | 0.0 | 0.0 | 0.0 | 0.0 | 0.0 | 0.0 | 0.0 | 0.0 | 0.0 | 0.0 |
| VIRULENCE : PROTEASES  VIRULENCE : PROTEASES | *aur* | aureolysin | 100.0 | 88.9 | 100.0 | 100.0 | 100.0 | 100.0 | 100.0 | 0.0 | 100.0 | 100.0 | 87.5 | 100.0 |
|  | *aur OtherThan252* | aureolysin from other strains (not MRSA252) | 100.0 | 100.0 | 100.0 | 100.0 | 100.0 | 100.0 | 0.0 | 0.0 | 100.0 | 100.0 | 87.5 | 100.0 |
|  | *aur-MRSA252* | aureolysin from strain MRSA252 | 0.0 | 0.0 | 0.0 | 0.0 | 0.0 | 0.0 | 100.0 | 100.0 | 0.0 | 0.0 | 0.0 | 0.0 |
|  | *splA* | serine protease A | 100.0 | 100.0 | 100.0 | 100.0 | 100.0 | 100.0 | 0.0 | 0.0 | 100.0 | 100.0 | 12.5 | 0.0 |
|  |  |  |  |  |  |  |  |  |  |  |  |  |  |  |
|  | *splB* | serine protease B | 100.0 | 100.0 | 100.0 | 100.0 | 100.0 | 100.0 | 0.0 | 0.0 | 100.0 | 100.0 | 100.0 | 0.0 |
|  | *splE* | serine protease E | 83.3 | 0.0 | 100.0 | 100.0 | 85.7 | 100.0 | 100.0 | 0.0 | 0.0 | 100.0 | 12.5 | 0.0 |
|  | *sspA* | glutamylendopeptidase | 100.0 | 100.0 | 100.0 | 100.0 | 100.0 | 100.0 | 100.0 | 100.0 | 100.0 | 100.0 | 100.0 | 100.0 |
|  | *sspB* | staphopain B, protease | 100.0 | 100.0 | 100.0 | 100.0 | 100.0 | 100.0 | 100.0 | 100.0 | 100.0 | 100.0 | 100.0 | 100.0 |
|  | *sspP* | staphopain A (staphopain A) protease | 100.0 | 100.0 | 100.0 | 100.0 | 100.0 | 100.0 | 100.0 | 100.0 | 100.0 | 100.0 | 100.0 | 100.0 |
| CAPSULE- AND BIOFILM-ASSOCIATED GENES | *cap1* | capsule type 1 | 0.0 | 0.0 | 0.0 | 0.0 | 0.0 | 0.0 | 0.0 | 0.0 | 0.0 | 0.0 | 0.0 | 0.0 |
|  | *cap5* | capsule type 5 | 0.0 | 100.0 | 0.0 | 100.0 | 0.0 | 100.0 | 0.0 | 0.0 | 0.0 | 100.0 | 0.0 | 100.0 |
|  | *cap8* | capsule type 8 | 100.0 | 0.0 | 100.0 | 0.0 | 100.0 | 0.0 | 100.0 | 100.0 | 100.0 | 0.0 | 100.0 | 0.0 |
|  | *icaA* | intercellular adhesion A | 100.0 | 100.0 | 100.0 | 100.0 | 100.0 | 100.0 | 100.0 | 100.0 | 100.0 | 100.0 | 75.0 | 100.0 |
|  | *icaC* | intercellular adhesion C | 100.0 | 100.0 | 100.0 | 100.0 | 100.0 | 100.0 | 100.0 | 100.0 | 100.0 | 100.0 | 100.0 | 0.0 |
|  | *icaD* | intercellular adhesion D | 100.0 | 100.0 | 100.0 | 100.0 | 100.0 | 100.0 | 100.0 | 100.0 | 100.0 | 100.0 | 75.0 | 100.0 |
|  | *bap* | surface protein involved in biofilm formation | 0.0 | 0.0 | 0.0 | 0.0 | 0.0 | 0.0 | 0.0 | 0.0 | 0.0 | 0.0 | 0.0 | 0.0 |
| MICROBIAL SURFACE COMPONENTS RECOGNIZING ADHESIVE MATRIX MOLECULES (MSCRAMM GENES | *bbp*  *clfA*  *clfB*  *cna*  *ebpS*  *fnbA*  *fnbB-COL+Mu5*  *map*  *sasG* | bone sialoprotein-binding protein  clumping factor A  clumping factor B  collagen-binding adhesion  cell surface elastin binding protein  fibronectin-binding protein A  fibronectin-binding protein B  major histocompatibility complex II analog protein  *Staphylococcus aureus* surface protein G | 100.0  100.0  100.0  100.0  100.0  100.0  100.0  66.7  100.0 | 88.9  100.0  100.0  0.0  100.0  100.0  77.8  100.0  100.0 | 100.0  100.0  100.0  0.0  100.0  100.0  100.0  100.0  0.0 | 100.0  100.0  100.0  0.0  100.0  100.0  100.0  100.0  100.0 | 100.0  100.0  85.7  0.0  100.0  100.0  100.0  100.0  100.0 | 100.0  100.0  100.0  0.0  100.0  100.0  50.0  100.0  0.0 | 100.0  100.0  100.0  100.0  100.0  100.0  100.0  87.5  0.0 | 50.0  100.0  100.0  100.0  100.0  100.0  100.0  100.0  0.0 | 100.0  100.0  100.0  0.0  100.0  100.0  100.0  100.0  100.0 | 100.0  100.0  100.0  0.0  100.0  100.0  100.0  100.0  100.0 | 75.0  100.0  100.0  100.0  100.0  100.0  75.0  100.0  0.0 | 100.0  100.0  100.0  100.0  100.0  100.0  100.0  0.0  0.0 |
| ACCESSORY GENE REGULATOR ALLELES (AGR  GROUP AFFILIATION) | *agrI* | accessory gene regulatory allele I | 0.0 | 0.0 | 100.0 | 100.0 | 0.0 | 100.0 | 0.0 | 100.0 | 0.0 | 100.0 | 50.0 | 100.0 |
|  | *agrII* | accessory gene regulatory allele II | 0.0 | 100.0 | 0.0 | 0.0 | 100.0 | 0.0 | 0.0 | 0.0 | 0.0 | 0.0 | 0.0 | 0.0 |
|  | *agrIII* | accessory gene regulatory allele III | 100.0 | 0.0 | 0.0 | 0.0 | 0.0 | 0.0 | 100.0 | 0.0 | 100.0 | 0.0 | 0.0 | 0.0 |
|  | *agrIV* | accessory gene regulatory allele IV | 0.0 | 0.0 | 0.0 | 0.0 | 0.0 | 50.0 | 0.0 | 0.0 | 0.0 | 0.0 | 100.0 | 100.0 |
